# A cross-sectional survey examining motivation and beliefs to participating in a web-based prospective cohort study on nutrition and health among individuals with a low socioeconomic status

**DOI:** 10.1186/s12889-020-08467-1

**Published:** 2020-03-17

**Authors:** Mélina Côté, Stéphanie Harrison, Annie Lapointe, Catherine Laramée, Sophie Desroches, Simone Lemieux, Benoît Lamarche, Ariane Bélanger-Gravel

**Affiliations:** 1 Institute of Nutrition and Functional Foods (INAF), Université Laval, Quebec City, Québec Canada; 2 Department of Information and Communication, Université Laval, Quebec City, Québec Canada; 3 Research Centre of the Quebec Heart and Lung Institute, Quebec City, Québec Canada

**Keywords:** Low socioeconomic status, Theory of planned behavior, Web-based studies, Beliefs, Recruitment, Retention

## Abstract

**Background:**

Prospective cohort studies may support public health efforts in reducing health inequalities. However, individuals with a low socioeconomic status (SES) are generally underrepresented in health research. This study aimed to examine the intention and determinants of intention of individuals with a low SES towards participation in a Web-based prospective project on nutrition and health (NutriQuébec) in order to develop recruitment and retention strategies.

**Methods:**

A cross-sectional survey based on the Theory of planned behaviour was conducted in the Province of Québec, Canada. Low SES individuals (high school or less and annual household income < $55,000 CAN) were recruited through a Web panel of a polling firm to assess intention, attitude, subjective norm and perceived behavioural control (PBC) towards participation in the NutriQuébec project. Linear regression and logistic regression analyses were conducted.

**Results:**

Mean age of respondents (184 women, 141 men) was 57.6 y (SD = 13.6). Attitude (*ß* = 0.54, 95%CI: 0.41–0.68) and PBC (*ß* = 0.50, 95%CI: 0.37–0.63) were significantly associated with intention. Participants who agreed that participating in the study would contribute to an improvement in 1) collective health (odds ratio [OR] = 2.15, 95%CI: 1.27–3.64) and in 2) one’s lifestyle habits (OR = 1.70, 95%CI: 1.04–2.78) were more likely to express positive intention compared to participants who did not agree with these statements. Participants who agreed to participate in the study even 1) in the absence of a financial incentive (OR = 1.43, 95%CI: 1.04–1.99) and even 2) if the completion of questionnaires took up to two hours (OR = 1.78, 95%CI: 1.27–2.48) were also more likely to express high intention. Receiving a personalized brief health assessment (OR = 1.61, 95%CI: 1.13–2.30) and the use of simple questions in the questionnaires (OR = 1.54, 95%CI: 1.05–2.25) were facilitating factors associated with high intention. Participants believing that participation would be too time-consuming were less likely to have positive intention (OR = 0.57, 95%CI: 0.43–0.75).

**Conclusions:**

The development of a positive attitude and a high PBC towards participation in the NutriQuébec project will be necessary to obtain representative data of low SES adults.

## Background

A clear gradient in health was recently observed in Canada among the more socially disadvantaged individuals [1]. It was observed that individuals with a lower socioeconomic status (SES) are more likely to adopt unhealthy lifestyle habits like smoking and being sedentary [2–4]. They are also more likely to have low quality diets and to face food insecurity [1, 3]. Consequently, low SES groups are more likely to develop chronic diseases, such as cardiovascular diseases and type 2 diabetes, and to have a lower life expectancy [1, 3]. Health inequalities are the result of interactions between multiple factors, including living conditions, psychosocial factors, health behaviours and genetic factors [1]. Despite this higher burden of diseases, individuals with a low SES do not always benefit from medical advances; this situation potentially leading to an increase in inequalities within the population [5]. Hence, health inequalities are a significant public health concern and reducing them represents a top priority [1, 6].

Prospective cohort studies may support public health efforts in reducing health inequalities by providing knowledge about the causes of these inequalities [6]. Such studies may also provide ongoing monitoring of the population’s health in the context of implementing and evaluating public health policies [1]. However, these studies, traditionally conducted by phone, postal mail or face-to-face interviews, can be burdensome and costly. Therefore, Web-based cohort studies raised enthusiasm in recent years due to their lower cost and time demand as well as the quality of data [7–9]. Web versions of a sociodemographic questionnaire [10], an anthropometric questionnaire [11] and a 24 h dietary recall [12] were validated in the *NutriNet-Santé France* prospective e-cohort study. Researchers concluded that data obtained from the Web questionnaires were of equal or superior quality when compared to data obtained from the paper-and-pencil versions of these questionnaires. Moreover, Web-based studies allow for an easier access to a larger number of potential participants, eliminating geographical boundaries and favoring easier access to hard-to-reach populations [9, 13–15]. In general, Internet access is now less perceived as a barrier to participate in Web-based studies [14–16] and can even be perceived as a facilitating factor. Indeed, in the *NutriNet-Santé France* study*,* half of the participants reported that they would not have participated in the project if it had not been conducted on the Internet [16]. On the other hand, the use of Web-based methods for data collection in health surveys and cohort studies remains a challenge. The European Health Interview Survey (EHIS) has shown that the use of mixed methods for data collection yielded a higher participation rate than the use of Web-based methods only [17]. Furthermore, although the majority of families living with an income lower than $20,000 have access to Internet in the context of the present study, a digital divide still exists in societies [18]. Low SES individuals still report lower access to Internet in their household [18] and, in some cases, have lower levels of health literacy and computer literacy due to lower education levels [19]. Considering the growing popularity of Web-based cohort studies and surveillance efforts, implementing strategies to maximize participation of individuals with a low SES in such studies is critical. Moreover, since individuals with a low SES are generally underrepresented in health studies [20, 21], recruitment and retention strategies must be adapted to encourage participation among these populations.

Epidemiological and surveillance research aimed at addressing social inequalities in health does not come without challenges. Indeed, transportation and time constraints [22–24], residential instability [22, 25], wrong numbers, disconnected phones and lack of answering machines [13, 22, 25], inability to reach potential participants at initial contact [25], economic constraints [23], mistrust of the Government and research, fear of exploitation as well as lack of knowledge [13, 23, 24] have all been identified as barriers to participating in studies among hard-to-reach populations such as those with a low SES, minority groups, and vulnerable populations. Hard-to-reach individuals are also often excluded from longitudinal studies because of acute medical or psychological conditions, or because of behavioural and social factors like alcoholism and drug abuse [23]. However, the barriers reported in these studies were not documented in the context of participation in Web-based prospective studies and were not conducted according to a validated theoretical framework. This is key because the use of theoretical frameworks to identify facilitators and barriers help in the development of more meaningful messages aimed at promoting the targeted behaviours (i.e., the participation in Web-based studies) [26]. To date, the very few studies that have used a validated theoretical framework to examine barriers towards participation in Web-based surveys were conducted among students or in the general population [27, 28]. Thus, the perceptions and beliefs of individuals with a low SES towards participation in prospective cohort studies remain essentially unknown.

According to the Theory of Planned Behaviour (TPB), the adoption of a given behaviour is determined by individuals’ intention (or motivation) to perform the behaviour. In turn, intention is influenced by individuals’ attitude (behavioural beliefs; advantages/disadvantages), the subjective norm (normative beliefs; approval/disapproval of significant others) and perceived behavioural control (PBC; control beliefs; facilitators/barriers) [29]. A meta-analysis reported that the TPB could explain 19.3% of the variance of several health-related behaviours and 44.3% of the variance of intention [30]. The TPB showed moderate to strong prediction of intention and behaviours across several health domains [31]. In addition, studies have previously used the TPB to examine beliefs related to participation in Web-based surveys [27, 28, 32]. Other studies based on the TPB have also demonstrated how beliefs related to one’s health can impact their decision to participate in health programs [33, 34]. Given the strong predictive validity of the TPB [29] and its recognition as a rigorous tool to examine behaviours related to participation in Web-based surveys, this theoretical framework was used in the present study.

A Web-based prospective study providing ongoing monitoring of the population’s health could allow evaluating public health policies as well as identifying and addressing health inequalities. Since individuals with a low SES are generally underrepresented in health studies, recruitment and retention strategies must be adapted to encourage participation among these populations in this type of research project. To support the development of effective strategies to recruit and retain individuals with a low SES, the aim of this study was to examine the determinants of intention and associated beliefs among individuals with a low SES toward participation in a Web-based prospective study, the NutriQuébec project, using the TPB [29].

## Methods

### Design and sampling

The NutriQuébec project is a Web-based, prospective study aimed at examining the impact of nutrition-related public health policies and action plans on the diet of the Québec adult population in Canada. More broadly, the NutriQuébec project aims to examine the associations between diet and health outcomes. To do so, male and female adults (18 years of age or more) are invited to complete a series of yearly core questionnaires through the Web, assessing dietary habits and other lifestyle habits (e.g., physical activity, sedentary behaviours, sleep, etc.), sociodemographic characteristics and general health. The time required to complete the core questionnaires on a yearly basis was estimated to two hours (unpublished data). Participants have a one-month period to complete the set of questionnaires. Participants may be invited to complete additional questionnaires on other nutrition-related issues between each yearly core measurements. A brief personalized assessment of dietary habits is returned yearly to each participant.

**Fig. 1:**
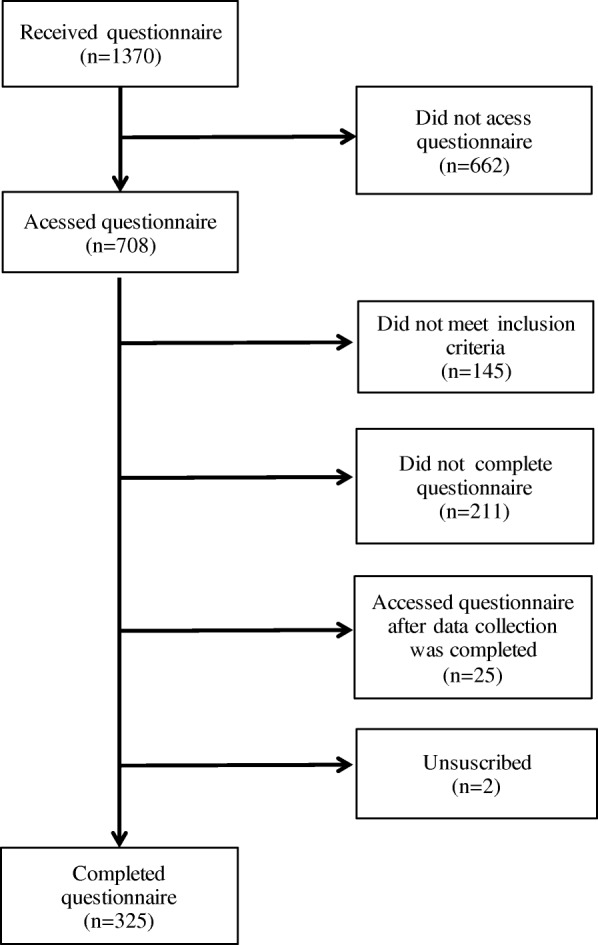
The flow diagram of study participation

A cross-sectional survey was conducted throughout the Province of Québec, Canada (sixteen administrative regions) among adults with a low SES. Respondents were recruited through a Web panel of a polling firm. The polling firm uses a pool of panelists that were randomly recruited among the Québec population to complete online surveys. The polling firm then sends a request to fill out the survey questionnaire to panelists corresponding to the inclusion criteria. The inclusion criteria were 1) to be able to complete a questionnaire in French, 2) to reside in the Province of Québec 3) to have low education (high school or less), and 4) to have low income (gross annual household income < $55,000 CAN). Overall, 1370 individuals were invited by the polling firm to complete the survey questionnaire (Fig. 1). Among those, 708 individuals accessed the questionnaire but 383 participants were excluded from the study because they did not meet the inclusion criteria (*n* = 145), accessed the questionnaire after data collection was completed (*n* = 25), unsubscribed from the polling firm panel (*n* = 2) or stopped the questionnaire while completing it (*n* = 211). Therefore, the data of 325 respondents were retained for analysis. The survey questionnaire was completed by participants on the Internet between the 11th and 20th of October 2018 and lasted approximately 15 min (Supplementary file 1). Respondents gave their implicit and anonymous consent by answering the questionnaire. The survey was approved by the Ethics Committee of Université Laval (2018–042/07-06-2018).

### Measurement

The survey questionnaire was developed for the purposes of the present study. It was developed following Ajzen’s guidelines for developing a TPB questionnaire [29] and included the behavioural and control beliefs identified in a preliminary elicitation study [32]. Briefly, four semi-structured focus groups, each comprising seven French-speaking adults, took place to identify behavioural beliefs, normative beliefs and control beliefs towards a hypothetical participation in the NutriQuébec project. The participants included in the focus groups were recruited through non-profit organizations (food banks, community organizations) known to serve populations living in deprived sectors of Québec City. Thereafter, two coders conducted an independent content analysis to classify the respondent’s answers in themes and to identify salient beliefs. The final classification of the beliefs was determined by consensus between the two coders. A third coder was involved when the two coders disagreed on some classifications. Since the aim of the present study was to develop strategies to optimize the recruitment and retention of individuals with a low SES in the NutriQuébec project, only beliefs that were the most relevant in the context of this project were included in the survey questionnaire. For example, although no or limited access to Internet was identified as a salient barrier to participation, this specific belief was not assessed in the survey because providing alternative methods such as sending a paper-and-pencil questionnaire by postal mail or providing direct support for Internet access was not considered in the NutriQuébec project because of feasibility issues. Similarly, factors that were out of the research team’s control (e.g., illness, little or no knowledge about Internet, etc.) or that were mentioned in only one out of the four focus groups were not included in the survey questionnaire. Finally, although budget constraints hinder the research team from offering monetary incentives or gift cards to participants, this was assessed anyways in order to grasp interest in participation if no incentive was offered. Before launching the survey, the questionnaire was evaluated by six experts in the fields of behaviour change, health communication, nutrition, and epidemiology to validate the clarity and the intelligibility of the questions.

Intention was assessed using three questions (Cronbach α = 0.81) with the following items: 1) “*I intend to participate in the NutriQuébec project*”, 2) *The chances that I participate in the NutriQuébec project are …* and, 3) *I will participate in the NutriQuébec project*. The answer choices varied from very unlikely to very likely and from very low to very high. Attitude was assessed using three questions (Cronbach α = 0.89): For me, participating in the NutriQuébec project would be … 1) *very unpleasant/very pleasant, 2) very useless/very useful and, 3) very unsatisfying/very satisfying*. Behavioural beliefs (five advantages and one disadvantage) were assessed using six questions. Injunctive norm was assessed using two questions with the following items: 1) *The most important people to me would think that I should participate in the NutriQuébec project* and 2) *If I were to participate in the NutriQuébec project, most people who are important to me would … strongly disagree/strongly agree.* Descriptive norm was assessed using one question: *Many people I know may be interested in participating in the NutriQuébec project*. Subjective norm represented the composite mean score of these three items and the Cronbach alpha was 0.66. Normative beliefs were not assessed in the present study questionnaire because no modal normative beliefs were identified during the preliminary elicitation study. PBC was assessed using three questions: 1) *I feel that I am capable of participating in the NutriQuébec project*, 2) *I am confident that I can overcome any obstacles that may prevent me from participating in the NutriQuébec project and*, 3) *For me, participating in the NutriQuébec project would be … very difficult/very easy* (Cronbach α = 0.82). Control beliefs were assessed using five questions (three facilitators and two barriers; see the Additional File for detailed description of the beliefs assessed). All psychosocial variables were measured using answer choices on five-point Likert or semantic scales. Participants also answered questions pertaining to their sociodemographic and socioeconomic position (sex, age, administrative area, education, income), Internet access at home as well as perceived computer skills. Perceived computer skills were assessed on a five-point Likert scale using the following question: *How would you rate your ability to browse the Internet?*

### Statistical analysis

Sample size estimations were based on a study by Rashidian et al., who determined the number of participants to conduct sufficiently powered TPB studies [35]. Continuous variables are described as means and standard deviations while categorical variables are described as proportions. Data were analysed using a stepwise approach. First, multivariate linear regression models were used to identify how the three TPB constructs were associated with intention to participate in NutriQuébec, as suggested by Fishbein and Yzer [36]. We then examined, using multivariate models, associations between behavioural and control beliefs and their associated TPB constructs. This analysis was restricted to the TPB constructs that showed significant associations with intention, as identified in the first step. In all univariate and multivariate analyses, effect sizes are reported as standardized betas (*ß*). Finally, logistic regression analyses were used to identify the behavioural and control beliefs that showed the strongest associations with intention to participate in NutriQuébec project. In this analysis, odds ratios (OR) reflected the likelihood of having a positive intention to participate, using negative intention as a reference (OR = 1.0). For that purpose, intention score was dichotomized with mean scores > 3 representing positive intention and mean scores ≤3 representing neutral or negative intention. TPB constructs and behavioural and control beliefs were treated as continuous variables in all analyses. All models were adjusted for sex, age, perceived ability to browse the Internet and for previous participation in studies on lifestyle habits conducted on the Internet. SAS Studio (v 3.71) was used for all analyses and *p-values* below 0.05 (two-tailed) were set to indicate the level of statistical significance.

## Results

### Participants’ characteristics

Sample size estimations determined that 148 participants were minimally needed to conduct the study. The sample comprised 184 women and 141 men with a mean age of 57.6 years (SD = 13.6). Sociodemographic and socioeconomic characteristics of the participants are presented in Table 1. The majority of participants had a high school degree as the highest education level attained (89%), reported being moderately competent, very competent or expert in browsing the Internet (86%), and had access to Internet at home (98%).

**Table 1:** Sociodemographic and Socioeconomic Characteristics of the Sample

Variables	
**Age (years), mean (SD)**	57.6 (13.6)
	**N (%)**
**Gender**	
Male	141 (43)
Female	184 (57)
**Education**	
None	36 (11)
High school or equivalent	289 (89)
**Annual household gross income**	
0-$15,000 CAN	38 (12)
$15,000- $24,999 CAN	67 (20)
$25,000- $34,999 CAN	84 (26)
$35,000- $54,999 CAN	136 (42)
**Internet access at home**	
Yes	320 (98)
No	5 (2)
**Computer skills**	
Not at all competent	4 (1)
Hardly competent	42 (13)
Moderately/rather competent	179 (55)
Very competent	82 (25)
Expert	18 (6)

### Linear regression analysis linking constructs with intention

The multivariable linear regression analysis revealed that attitude (*ß* = 0.54, 95% CI: 0.41–0.68) and PBC (*ß* = 0.50, 95% CI: 0.37–0.63) were significantly associated with intention score, explaining 67% of its variance. Subjective norm was not associated with intention to participate in the NutriQuébec project (*ß* = 0.05, 95% CI: − 0.05-0.16). Similar results were obtained among men and women when analyzed separately (data not shown).

### Linear regression analysis linking behavioural and control beliefs with corresponding constructs

Table 2 shows the associations between respondents’ behavioural and control beliefs and attitude and PBC respectively. The behavioural beliefs (independent variables) that were significantly associated with attitude (dependant variable) in multivariate analyses were: 1) contributing to improving collective health (*ß* = 0.23, *p* = 0.0003), 2) having the opportunity to improve one’s lifestyle habits (*ß* = 0.30, *p* = < 0.0001), and 3) the fact that completing the survey questionnaires would be too time-consuming (*ß* = − 0.18, *p* = < 0.0001). The control beliefs (independent variables) that were significantly associated with PBC (dependant variable) were: 1) receiving a personalized brief health assessment (*ß* = 0.14, *p* = 0.0006), 2) the use of simple questions (*ß* = 0.13, *p* = 0.0062), 3) participating in the research project even without financial incentives (*ß* = 0.11, *p* = 0.0011), and 4) participating in the research project even if the completion of the questionnaires would take two hours (*ß* = 0.19, *p* = < 0.0001).

**Table 2:** Multivariate Analyses of Behavioural and Control Beliefs Associated with Attitude and PBC Towards Participation in NutriQuébec

Items	Standardized ß	***P***-value*	95% CI
**Behavioural beliefs associated with attitude**			
*Advantages*			
Contributing to improving collective health	0.23	0.0003	0.11–0.35
Having the opportunity to improve one’s lifestyle habits	0.30	< 0.0001	0.17–0.43
Having the opportunity to improve the family’s lifestyle habits	0.02	0.73	−0.09 - 0.13
Learning new knowledge on health	0.05	0.46	−0.09 - 0.19
Contributing to advancing science	0.08	0.15	−0.03 - 0.19
*Disadvantages*			
Too time-consuming to complete the questionnaire	−0.18	< 0.0001	−0.24 - -0.12
**Model R**^**2**^	**58.8**		
**Control beliefs associated with PBC**			
*Facilitating factors*			
Receiving a personalized brief health assessment	0.14	0.0006	0.06–0.22
The use of simple questions	0.13	0.0062	0.04–0.23
Anonymity	−0.02	0.6597	− 0.10 - 0.06
*Barriers*			
Participating even without financial incentives	0.11	0.0011	0.04–0.18
Participating even if the completion of the questionnaires would take two hours	0.19	< 0.0001	0.11–0.26
**Model R**^**2**^	**45.2**		

*p-values and 95%Cl were found using linear regression models

### Logistic regression analysis of intention to participate in the NutriQuébec project

**Fig. 2:**
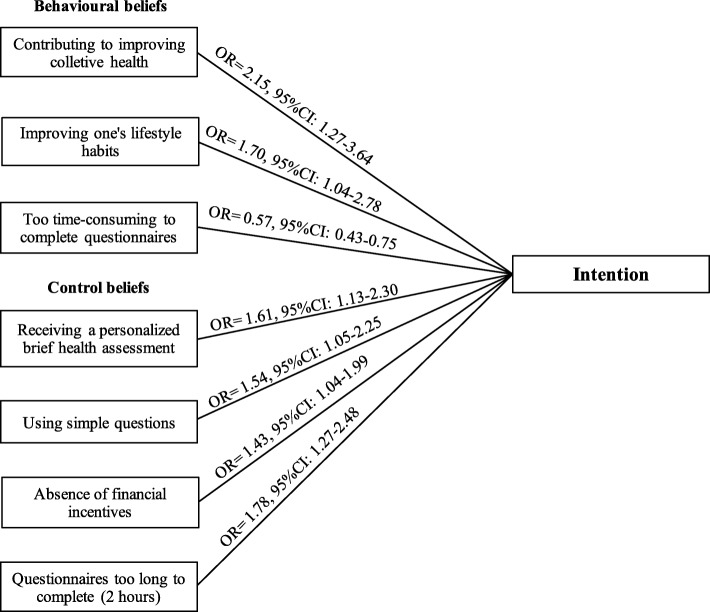
Beliefs associated with positive intention towards participating in the Web-based NutriQuébec project

Figure 2 shows the results of the logistic regression analysis aimed at identifying beliefs that are associated with intention to participate in the NutriQuébec project. This analysis showed that participants who scored favorably for behavioural and control beliefs associated with attitude and PBC respectively also expressed a greater likelihood to participate in NutriQuébec than participants with negative scores for behavioural and control beliefs.

## Discussion

NutriQuébec is a Web-based prospective study on diet and health among adults from the Province of Québec, Canada. The objective of this study was to examine the determinants of intention and its associated beliefs towards participation in the NutriQuébec project, targeting hard-to-reach individuals with a low SES. The results showed that attitude and PBC were both associated with potential participation in the NutriQuébec project and highlighted some key beliefs that should be considered to maximize recruitment and retention of individuals with a low SES. These study results are of most relevance to the NutriQuébec project. These findings could also be of interest to other prospective Web-based studies wanting to optimize recruitment and retention strategies among individuals with a low SES, although not being necessarily generalizable to all other Web-based studies because of the specific elements of the NutriQuébec project.

Results suggest that favouring a positive attitude and a high PBC towards participation will be essential, whereas developing messages around some form of social approval is unlikely to facilitate recruitment of individuals with a low SES. This is consistent with the results from our preliminary elicitation study, in which no salient normative beliefs clearly emerged towards potential participation in the NutriQuébec project among adults living in deprived urban areas [32]. A possible reason why subjective norm does not result in a significant determinant of intention could be the fact that the behaviour, participating in the NutriQuébec project, does not directly impact a person’s environment or significant others. Similarly, participants in a study evaluating the intention to consult a blog delivered by a registered dietitian to improve their dietary habits reported that this action did not require the approval of others, since it was considered rather personal [37].

Our results are, however, at odds with data from previous studies on this topic. The study by Farmer et al. [38], which examined the TPB determinants of intention to participate in a medical research project among low SES and African American women, showed that attitude, PBC and perceived social norms were all associated with intention. However, it is noteworthy that the social norms related themes were trust (or mistrust) in researchers and negative perceptions about the behaviour of researchers rather than focussing on others’ approval/disapproval [38]. Moreover, this study was not about participation in Web-based surveys. The study by Heerwegh et al. [27] also reported that positive attitudes, a higher degree of PBC and a positive subjective norm were all associated with a higher intention to participate in a Web-based survey among the general population. Similarly, Bosnjak et al. [28] observed that PBC best predicted intention to participate in a Web-based survey among undergraduate students, followed by attitude and subjective norm. Differences in results between our and previous study results may be explained to a large extent by the nature of the study populations with regards to SES. To our knowledge, the present study is the first to identify the determinants of intention to participate in a Web-based study on diet and health among individuals with a low SES specifically. Discrepancies between results also highlight a basic premise of the reasoned action approach (from which the TPB relies): beliefs and determinants of a behaviour are highly context-dependant and should always be assessed when attempting to intervene in a target population [39]. Nonetheless, results seem to be coherent with the fact that participation in a study relies mostly on attitude and PBC.

The positive behavioural beliefs (or perceived advantages) that were significantly associated with a positive attitude and intention towards participation in the Web-based NutriQuébec project pertained to the potential contribution to improving collective health and one’s lifestyle habits. Altruistic motivation to participate in research projects has been reported previously [16, 38, 40, 41]. For instance, African American and low SES women mentioned in focus groups that they would participate in a research project if it could possibly benefit others in the future [38]. Another study on the motives towards participation in a Web-based prospective study reported that the majority of participants took part in the research project to contribute to advancing public health research [16]. Contributing to improving the population’s health and the environment was also identified as benefits to participating in a prospective cohort study [41] as well as in a study regarding the creation of a research database comprising personal, clinical and genetic information [40]. Interestingly, the second positive behavioural belief associated with participation in the NutriQuébec project pertained to the opportunity to improve one’s health. This is in line with previous studies on altruism, which highlighted that pure altruism is not always observed and that individuals expect some form of reward in exchange for their time and participation [38, 42]. This suggests that framing participation in the Web-based NutriQuébec project as an altruistic act may be a promising strategy to recruit individuals with a low SES. However, persuasive messages may also need to highlight the potential opportunity to learn more about one’s health and even improve it. The fact that obtaining a personalized brief health assessment was among the significant discriminant facilitating factors further supports this assertion. The recruitment and probably the retention of participants with a low SES in the NutriQuébec project are therefore likely to be facilitated by providing a brief health assessment as a participation reward. Similar findings were reported by Yamamoto et al., where participants with a high level of motivation to participate in a study mentioned that having access to their own results was a motivation factor [41]. Nevertheless, providing individualized feedback on diet and health raises concerns in the context of conducting a prospective study aimed at examining the associations between the population’s health and the impact of policies on health since providing a health assessment might be considered as an intervention by itself [43]. Thus, providing feedback needs to be balanced between participation reward and satisfaction and behaviour change motivation to limit potential bias in estimations. Strategies to control for potential impacts of the feedback mechanisms on participants’ behaviours over time may also need to be implemented to ensure that they can be captured and adjusted for in the analyses.

The burdensomeness of taking time to fill out numerous questionnaires was a negative behavioural belief (or a disadvantage) associated with participating in the NutriQuébec project. Similarly, it was observed from a longitudinal study with weekly Web surveys over 2.5 years that some participants found the long-term and repetitive nature of surveys tedious [44]. The time burden associated with participation of low SES or minority populations in studies has also been documented elsewhere [22, 23, 45], suggesting that the idea of being considerably committed to a study discourages participants and may increase attrition over time. Considering the fact that prospective studies necessarily involve repeated observations/measures over long periods of time, strategies in data collection must be adopted to make sure that participants perceive more benefits than disadvantages. Retention strategies in longitudinal studies include the reduction of participant burden (convenience, simplicity, not feeling judged, etc.) [46] and taking into account participant suggestions regarding data collection [47].

Participants who declared that they would participate in the research project even in the absence of monetary incentives expressed a higher degree of PBC and intention. This suggests that providing a financial incentive to increase participation in a Web-based longitudinal study may not be necessary among individuals with low SES. This is consistent with findings by Edwards et al., which showed no evidence that a monetary incentive encouraged participants to complete Web-based surveys [48]. Instead, they reported that offering a non-monetary incentive increased the odds of completing a Web-based survey by two-folds [48]. It should be noted that findings from this Cochrane Review were not necessarily drawn from studies conducted only among SES populations. This is also consistent with our elicitation study, in which all focus groups expressed an interest in non-monetary incentives, like receiving a health assessment [32]. Likewise, results from the present study showed that receiving a personalized brief health assessment was associated with high PBC and high intention to participate in the NutriQuébec project. On the other hand, other studies have shown that providing a financial incentive was effective to encourage low-income, unemployed or hard-to-reach individuals to participate in a study [49–51]. Yu et al. reported that the use of a modest monetary incentive ($10) in a longitudinal study increased the number of returned surveys [52]. Taken together, these results suggest that the effect of a monetary incentive on recruitment and retention of participants remains uncertain, particularly for long surveys, and more studies on this topic are needed to clarify the impact of such an approach on participation in Web-based research projects.

Studies have often reported an inverse association between the length of the questionnaires and response rate [48, 53–55]. Inversely, results from another study showed that longer questionnaires were associated with higher participation rates, which could possibly be explained by the fact that a greater involvement in the study seemed to increase interest in research participation [56]. Participation in epidemiological studies has decreased in the past decades [46], emphasizing the importance of identifying underlying factors, including questionnaire length. Considering the fact that the NutriQuébec project requires completing questionnaires once a year, it was important to know if the length of these questionnaires would be a barrier to participate in the study for individuals with a low SES. Our results suggest that participants who would participate in the research project even if the questionnaires took two hours to complete had a high degree of PBC and high intention. Allowing participants to complete questionnaires over an extended period of time rather than during the predefined one-month window of time may decrease the perception that participation is burdensome and increase self-efficacy toward this behaviour. However, at study onset, it was decided not to modify the period to fill out all questionnaires to minimize time disparity in the measurement of the various health behaviours.

The use of simple questions was significantly associated with high PBC and high intention towards participation in the NutriQuébec project. This result highlights the significant need to address literacy issues to recruit and retain individuals with a low SES in prospective research projects. Consistent with this, a study reported that individuals with low literacy were more likely to prefer simple sentences when presented with six different descriptions for each predetermined research term (‘randomisation’, ‘informed consent’, ‘confidentiality’ and ‘why carry out research?’) [57]. Low literacy is more prevalent among adults with a low SES. Indeed, among the Québec adults (18–65 years) who do not have a high school diploma, unemployed and low literacy persons are overrepresented [58]. Additionally, a study reported that adults with low health literacy and numeracy were significantly less interested in participating in research projects, most probably because of the difficulties associated with understanding consent forms and perceived risks pertaining to the project as well as the skills needed to complete survey questionnaires [59]. To help recruit individuals with a low SES among the Québec population, efforts need to be made to simplify the questionnaires and test them for clarity within low SES groups. Efforts will also be made to present and explain the study in simple terms on the NutriQuébec website.

### Strengths and limitations

As indicated above, this is the first study, to our knowledge, that examined determinants of intention of participating in a Web-based prospective cohort study as well as the influence of behavioural and control beliefs on this intention among a sample of adults with a low SES. The TPB was used as a validated framework for the basis of this study, which is another strength because of its strong predictive capacity. Also, participants were recruited from sixteen different administrative regions in the Province of Québec, Canada, which suggests a good representation of individuals with a low SES across the Province. On the other hand, this study was only conducted in French, which may not represent the beliefs of English-speaking Quebecers, as well as those of immigrants or Indigenous people who cannot complete the survey questionnaire in French. Findings may also not be representative of all low SES individuals in Québec considering that the vast majority of participants reported having access to Internet and being competent using the Internet, which is most likely related to recruitment via a Web panel of a polling firm. Additionally, given the relatively low response rate, a selection bias cannot be ruled out. Indeed, it is possible that the individuals who participated in the study had a greater interest in nutrition and therefore were more prone to having a higher intention of participating in the NutriQuébec project as well as to perceiving more advantages and fewer barriers. Findings may also not be generalizable to all prospective Web-based studies considering the specific elements of the NutriQuébec project. Lastly, the cross-sectional nature of the study is also a limitation. Future studies would benefit from identifying whether intention leads to behaviour within this population.

## Conclusions

This study examined for the first time the intention of participating in a Web-based prospective cohort study among adults with a low SES, as well as the influence of behavioural and control beliefs on intention. Using the TPB, we observed that favouring a positive attitude and a high PBC towards participation in a Web-based project are likely to be key factors facilitating recruitment among adults with a low SES. Furthermore, altruism, improving lifestyle habits and receiving a health assessment were significant behavioural and control beliefs that should be leveraged in the recruitment and retention strategies to encourage participation in the NutriQuébec project. Questionnaires should also be as simple and short as possible to favour participation of adults with a low SES. These results are of importance for future Web-based prospective studies in order to adapt recruitment and retention strategies to help capture data from all groups of the population, particularly those that are more socially disadvantaged.

## Supplementary information

**Additional file 1: Supplementary File 1.** The TPB Survey Questionnaire.

## Data Availability

The datasets used and/or analyzed during the current study are available from the corresponding author on a reasonable request.

## Abbreviations

PBC: Perceived behavioural control
SES: Socioeconomic status
TPB: Theory of planned behaviour
