# Supplementary material for: A cross-sectional survey examining motivation and beliefs to participating in a web-based prospective cohort study on nutrition and health among individuals with a low socioeconomic status
Source: BMC Public Health. 2020 Mar 17;20:348. doi: 10.1186/s12889-020-08467-1 (PMC7079419; doi:10.1186/s12889-020-08467-1)
Supplement: Supplementary file 1 — Additional file 1: Supplementary File 1. The TPB Survey Questionnaire. [file 12889_2020_8467_MOESM1_ESM.docx]

**Supplementary File 1. The TPB Survey Questionnaire**

*A group of researchers coming from everywhere in the Province of Québec are preparing the* ***NutriQuébec*** *project.* *This extensive project aims to examine the associations between lifestyle habits and the population’s health. Quebecers will be invited to participate by completing the questionnaires on the Web every year for many years. It will be the greatest study on lifestyle habits and health ever realized in the Province of Québec until now.*

*The questionnaires will be about:*

- *Diet*
- *Physical activity*
- *Lifestyle habits (ex: sleep, smoking, etc.)*
- *Personal characteristics (ex: income, education, etc.)*
- *Health in general*

*The NutriQuébec project will include many questions* ***to complete over a one-month period****. We estimate that it will require about* ***2 hours*** *to complete all questionnaires. The participants will be able to complete the questionnaires whenever they wish. Before starting the study, the researchers wish to know your opinion on the different aspects of the NutriQuébec project. That is why we are inviting you today to complete this survey.*

1. In which administrative region do you live?
   - Bas-Saint-Laurent
   - Saguenay-Lac-Saint-Jean
   - Capitale-Nationale
   - Mauricie
   - Estrie
   - Montréal
   - Outaouais
   - Abitibi-Témiscamingue
   - Côte-Nord
   - Nord-du-Québec
   - Gaspésie-Îles-de-la-Madeleine
   - Chaudière-Appalaches
   - Laval
   - Lanaudière
   - Laurentides
   - Montérégie
   - Centre-du-Québec
   - I do not know my administrative region
   - I do not live in the Province of Québec
2. Are you 18 years or older?

- Yes
- No

***The participants of the NutriQuébec project will be invited to complete the same questionnaires every year for many years.***

1. I intend to participate in the NutriQuébec project.

|  | Very  unlikely |  | Somewhat unlikely |  | Neither likely nor unlikely |  |  | Somewhat  likely |  | Very  likely |
| --- | --- | --- | --- | --- | --- | --- | --- | --- | --- | --- |
|  | □ |  | □ |  | □ |  |  | □ |  | □ |

1. I would participate in the NutriQuébec project if…

|  | Very  unlikely | Somewhat unlikely | Neither likely nor unlikely | Somewhat  likely | Very  likely |
| --- | --- | --- | --- | --- | --- |
| … I would receive a personalized brief health assessment (my results from the questionnaires). | □ | □ | □ | □ | □ |
| … the questions would be simple. | □ | □ | □ | □ | □ |
| … my answers to the questionnaires would remain anonymous. | □ | □ | □ | □ | □ |

1. The NutriQuébec project will include multiple questions to complete over a one-month period. We estimate that about 2 hours are required from participants to complete the set of questions. The researchers wish to identify the best way to administer these questions in order to facilitate the work of the participants.

Which of the three following options do you prefer?

- The questions are found in only one questionnaire (duration: 2 hours)
- The questions are shared in 5 questionnaires (duration: 25 minutes each)
- The questions are shared in 12 short questionnaires (duration: 10 minutes each)

1. If I were to participate in the NutriQuébec project, it would take me too much time to answer the questionnaire.

|  | Strongly disagree |  | Somewhat disagree |  | Neither agree nor disagree |  |  | Somewhat  agree |  | Strongly  agree |
| --- | --- | --- | --- | --- | --- | --- | --- | --- | --- | --- |
|  | □ |  | □ |  | □ |  |  | □ |  | □ |

1. The most important people to me would think that I should participate in the NutriQuébec project.

|  | Strongly disagree |  | Somewhat disagree |  | Neither agree nor disagree |  |  | Somewhat  agree |  | Strongly  agree |
| --- | --- | --- | --- | --- | --- | --- | --- | --- | --- | --- |
|  | □ |  | □ |  | □ |  |  | □ |  | □ |

1. I feel that I am capable of participating in the NutriQuébec project.

|  | Strongly disagree |  | Somewhat disagree |  | Neither agree nor disagree |  |  | Somewhat  agree |  | Strongly  agree |
| --- | --- | --- | --- | --- | --- | --- | --- | --- | --- | --- |
|  | □ |  | □ |  | □ |  |  | □ |  | □ |

1. Many people I know may be interested in participating in the NutriQuébec project.

|  | Strongly disagree |  | Somewhat disagree |  | Neither agree nor disagree |  |  | Somewhat  agree |  | Strongly  agree |
| --- | --- | --- | --- | --- | --- | --- | --- | --- | --- | --- |
|  | □ |  | □ |  | □ |  |  | □ |  | □ |

1. I am confident that I can overcome any obstacles that may prevent me from participating in the NutriQuébec project.

|  | Strongly disagree |  | Somewhat disagree |  | Neither agree nor disagree |  |  | Somewhat  agree |  | Strongly  agree |
| --- | --- | --- | --- | --- | --- | --- | --- | --- | --- | --- |
|  | □ |  | □ |  | □ |  |  | □ |  | □ |

1. For me, participating in the NutriQuébec project would be…

|  | Very unpleasant |  | Somewhat unpleasant |  | Neither pleasant nor unpleasant |  |  | Somewhat  pleasant |  | Very  pleasant |
| --- | --- | --- | --- | --- | --- | --- | --- | --- | --- | --- |
|  | □ |  | □ |  | □ |  |  | □ |  | □ |

|  | Very  useless |  | Somewhat useless |  | Neither useful nor useless |  |  | Somewhat  useful |  | Very  useful |
| --- | --- | --- | --- | --- | --- | --- | --- | --- | --- | --- |
|  | □ |  | □ |  | □ |  |  | □ |  | □ |

|  | Very unsatisfying |  | Somewhat unsatisfying |  | Neither satisfying nor unsatisfying |  |  | Somewhat  satisfying |  | Very  satisfying |
| --- | --- | --- | --- | --- | --- | --- | --- | --- | --- | --- |
|  | □ |  | □ |  | □ |  |  | □ |  | □ |

1. For me, participating in the NutriQuébec project would be…

|  | Very  difficult |  | Somewhat difficult |  | Neither easy nor difficult |  |  | Somewhat  easy |  | Very  easy |
| --- | --- | --- | --- | --- | --- | --- | --- | --- | --- | --- |
|  | □ |  | □ |  | □ |  |  | □ |  | □ |

1. The chances that I participate in the NutriQuébec project are…

|  | Very  small |  | Somewhat small |  | Neither big nor small |  |  | Somewhat  big |  | Very  big |
| --- | --- | --- | --- | --- | --- | --- | --- | --- | --- | --- |
|  | □ |  | □ |  | □ |  |  | □ |  | □ |

1. I would participate in the NutriQuébec project even if…

|  | Very  unlikely | Somewhat unlikely | Neither likely nor unlikely | Somewhat  likely | Very  likely |
| --- | --- | --- | --- | --- | --- |
| … no financial incentive would be offered. | □ | □ | □ | □ | □ |
| … the total duration for completing the questionnaires would be 2 hours. | □ | □ | □ | □ | □ |

1. I will participate in the NutriQuébec project.

|  | Very  unlikely |  | Somewhat unlikely |  | Neither likely nor unlikely |  |  | Somewhat  likely |  | Very  likely |
| --- | --- | --- | --- | --- | --- | --- | --- | --- | --- | --- |
|  | □ |  | □ |  | □ |  |  | □ |  | □ |

1. If I were to participate in the NutriQuébec project, this would…

|  | Very  unlikely | Somewhat unlikely | Neither likely nor unlikely | Somewhat  likely | Very  likely |
| --- | --- | --- | --- | --- | --- |
| … contribute to improving the Québec population’s health. | □ | □ | □ | □ | □ |
| … help me to improve my lifestyle habits | □ | □ | □ | □ | □ |
| … help to improve my family’s lifestyle habits | □ | □ | □ | □ | □ |
| … allow me to have new knowledge on health. | □ | □ | □ | □ | □ |
| … allow me to contribute to advancing science. | □ | □ | □ | □ | □ |

1. If I were to participate in the NutriQuébec project, most people who are important to me would…

|  | Strongly disapprove |  | Somewhat disapprove |  | Neither approve nor disapprove |  |  | Somewhat  approve |  | Strongly approve |
| --- | --- | --- | --- | --- | --- | --- | --- | --- | --- | --- |
|  | □ |  | □ |  | □ |  |  | □ |  | □ |

***In the NutriQuébec project, additional questionnaires could be sent to some participants a couple times per year.***

*The precise subject of these questionnaires will be determined progressively according to the research needs and current events. For example, if a tax on sugary beverages were to be implemented, some participants could be invited to complete a questionnaire about their opinion regarding this new tax.*

*The additional questionnaires would take a maximum of 20 minutes to complete.*

***For the following question, choose the answer that corresponds best to your opinion.***

1. If I would receive additional questionnaires, I would accept to complete them.

|  | Very  unlikely |  | Somewhat unlikely |  | Neither likely nor unlikely |  |  | Somewhat  likely |  | Very  likely |
| --- | --- | --- | --- | --- | --- | --- | --- | --- | --- | --- |
|  | □ |  | □ |  | □ |  |  | □ |  | □ |

***In the NutriQuébec project, participants will be invited to provide their health insurance number.***

*The main objective of the NutriQuébec project is to examine the associations between lifestyle habits and health. To do so, we will ask participants if they were diagnosed with illnesses by a health professional and if they take medication. We will also use the information from the participant’s medical record, via their health insurance number. This will allow us to have a valid and rigorous study. The medical information provided by the participant will be kept under high security and will never be transmitted to researchers or third parties, like insurance companies.*

***For the following question, choose the answer that corresponds best to your opinion.***

1. If I would be ensured that my medical data would stay confidential and securely kept, I would agree to provide my health insurance number.

|  | Very  unlikely |  | Somewhat unlikely |  | Neither likely nor unlikely |  |  | Somewhat  likely |  | Very  likely |
| --- | --- | --- | --- | --- | --- | --- | --- | --- | --- | --- |
|  | □ |  | □ |  | □ |  |  | □ |  | □ |

***Within the context of a second part of the NutriQuébec project, some participants would be invited to go to the research center to carry out different tests including, for example:***

*Weight and height measurement, waist circumference, blood pressure, etc.*

*Blood sample: cholesterol, blood sugar level, etc.*

*Note that parking or bus fees would be reimbursed.*

***For each question, choose the answer that corresponds best to your opinion.***

1. If I would be invited, I would agree to go to a research center to carry out some tests.

|  | Very  unlikely |  | Somewhat unlikely |  | Neither likely nor unlikely |  |  | Somewhat  likely |  | Very  likely |
| --- | --- | --- | --- | --- | --- | --- | --- | --- | --- | --- |
|  | □ |  | □ |  | □ |  |  | □ |  | □ |

1. If I would be offered a personalized health assessment (results of my tests), I would accept going to the research center to carry out some tests.

|  | Very  unlikely |  | Somewhat unlikely |  | Neither likely nor unlikely |  |  | Somewhat  likely |  | Very  likely |
| --- | --- | --- | --- | --- | --- | --- | --- | --- | --- | --- |
|  | □ |  | □ |  | □ |  |  | □ |  | □ |

1. By which communication channels the NutriQuébec advertisements would have the greatest chance to grab your attention? Please rank the following answer choices in order of preference (1= the one that you prefer the most, …, 7 = the one that you prefer the least).

|  | 1 | 2 | 3 | 4 | 5 | 6 | 7 |
| --- | --- | --- | --- | --- | --- | --- | --- |
| Television | □ | □ | □ | □ | □ | □ | □ |
| Social media (Facebook, Instagram, Youtube, etc.) | □ | □ | □ | □ | □ | □ | □ |
| Internet (other than social media) | □ | □ | □ | □ | □ | □ | □ |
| Newspaper | □ | □ | □ | □ | □ | □ | □ |
| Posters in public places (hospitals, community centers, etc.) | □ | □ | □ | □ | □ | □ | □ |
| The Publisac | □ | □ | □ | □ | □ | □ | □ |
| Postal mail | □ | □ | □ | □ | □ | □ | □ |

- None of these channels have a chance to grab my attention.

1. Who could motivate you to participate in the NutriQuébec project?

- A popular artist
- A sports personality
- A person known for his/her healthy lifestyle
- A participant in the NutriQuébec project
- The people in charge of the NutriQuébec project
- No one could motivate me to participate in the NutriQuébec project

1. Do you have an example of a popular artist?

|  |
| --- |

1. Do you have an example of a sports personality?

|  |
| --- |

1. Do you have an example of a person known for his/her healthy lifestyle?

|  |
| --- |

1. I have already participated in a study on lifestyle habits that consisted of completing many questionnaires on the Web.

- Yes
- No

1. What is your sex?

- Male
- Female

1. What is your age? _____________
2. What is the highest diploma, certificate or grade that you have completed?

- None
- High school diploma or equivalent (DES)
- Diploma of vocational studies (DEP)
- Collegial studies diploma or certificate (DEC)
- University certificate or diploma below a bachelor’s degree
- Bachelor’s degree
- University certificate or diploma above a bachelor’s degree
- Master’s degree
- Diploma in medicine, dentistry, veterinary medicine or optometry
- Earned doctorate
- I prefer not to answer

1. What is your annual gross household income (before taxes)?

- Less than $15,000
- $15,000-$24,999
- $25,000-$34,999
- $35,000-$54,999
- $55,000-$74,999
- $75,000-$99,999
- $100,000 or more
- I prefer not to answer

1. Do you have Internet access at home?

- Yes
- No

1. Do you have Internet access at work?

- Yes
- No
- Not applicable

1. If you have to complete questionnaires on the Web, which device(s) would you use?

- Computer
- Smartphone
- Tablet (ex: IPad)

1. How would you rate your ability to browse the Internet?

- Not at all competent
- Hardly competent
- Moderately/rather competent
- Very competent
- Expert
